# Evaluating the Association Between the Extent of Resection and Survival in Gliosarcoma

**DOI:** 10.7759/cureus.4374

**Published:** 2019-04-03

**Authors:** Fahad I Ahmed, Kalil G Abdullah, Joseph Durgin, Ryan D Salinas, Donald M O'Rourke, Steven Brem

**Affiliations:** 1 Neurosurgery, Hospital of the University of Pennsylvania, Philadelphia, USA

**Keywords:** gliosarcoma, high-grade glioma, gliosarcoma survival, extent of resection

## Abstract

Introduction: Gliosarcoma (GS) is a rare, malignant mixed tumor of the central nervous system with a median survival of approximately 13 months across multiple studies. Although the value of the extent of resection (EOR) has been confirmed as a prognostic survival factor in glioblastoma, no such association has been defined for GS. The goal of this study was to establish an association between EOR and survival and to determine if a threshold of resection exists for which a survival benefit is conferred in GS.

Methods: The authors identified 11 patients with histologically confirmed GS between January 2005 and January 2015, treated at the Hospital of the University of Pennsylvania. Clinical, radiographic, and outcome data were retrospectively reviewed. Volumetric analysis was completed using semi-automated segmentation to measure the change in contrast-enhancing material based on preoperative T1-contrast (T1c) and postoperative T1 & T1c magnetic resonance imaging (MRI) scans. A log-rank test was completed to confirm an association between EOR and survival, and a series of Kaplan-Meier curves were constructed to determine an EOR threshold. Univariate Cox proportional hazards model (CPHM) followed by multivariate CPHM was also completed to evaluate associations between the prognostic clinical and immunohistochemistry variables under consideration.

Results: Extent of resection categories were defined as gross total resection (GTR >95%), subtotal resection (STR 90%-95%), and partial resection (PR <90%). The median overall survival for the groups were as follows: GTR-17.3 months (n=4), STR-12.6 months (n=5), PR-4.3 months (n=2). A statistically significant association (p=05 level) was found between survival and the PR group with the GTR group as reference. Multivariate CPHM confirmed a statistically significant association between increased survival and age, preoperative Karnofsky Performance Status (KPS) scores, postoperative KPS scores, and KI-67 index. Serial Kaplan-Meier curves suggest a survival benefit with an EOR threshold of 94%.

Conclusion: This study agrees with previous correlations in glioblastoma EOR and prolonged survival. For patients undergoing surgical resection for GS, maximal surgical removal, when safely possible, should be attempted as it appears to translate to longer survival times.

## Introduction

Gliosarcoma (GS) is a rare form of glioblastoma with unique pathological indicators that has an increased propensity to metastasize outside of the cerebrum. It is a primary mixed tumor, consisting of both sarcomatous and glial elements. Median survival time is approximately 13 months [1]. Histopathology confirms approximately 2% of diagnosed high-grade gliomas to be GSs [2]. Consequently, knowledge of the disease is based on small retrospective studies and is generally limited.

In this study, we attempt to establish an association with the extent of resection (EOR) and survival in GS patients. In addition, we analyze immunohistochemistry characteristics to determine if an association with any biomolecular markers exists with increased survival.

## Materials and methods

Study design

Between January 2005 and January 2015, 11 patients who underwent index resection for GS at the Hospital of the University of Pennsylvania met inclusion data for this retrospective cohort study. None of the patients in our study underwent previous resection operations, nor did they have a previously identified low-grade tumor. Pathology review was completed based on the World Health Organization (WHO) guidelines to confirm all patients had high-grade GS. Clinical, radiographic, and outcome data were retrospectively collected from patient records. The institutional review board of the University of Pennsylvania (IRB #825635) approved this study.

Imaging analysis

Tumor volume was measured using a semi-automated segmentation procedure in the ITK-Snap software package (CBICA, Philadelphia, Pennsylvania, USA). The classification extension of the program was used to measure the contrast enhancing material based on preoperative T1-contrast (T1c) and postoperative T1 & T1c magnetic resonance imaging (MRI) scans. The extent of resection was calculated using the following formula: (preoperative tumor volume - postoperative tumor volume)/ preoperative tumor volume). The software extension required us to define tumor boundaries by marking regions as within or outside the tumor, allowing the algorithm to mark the tumor in an iterative process. Once the algorithm completed tumor marking, we reviewed the results and modified the segmentation as necessary to ensure proper results. This segmentation was completed without knowledge of patient outcomes.

Statistical analysis

We constructed Kaplan-Meier curves to show differing survival among EOR categories. A paired log-rank (Mantel-Cox) test was completed to determine statistical significance among survival times between EOR groups, using the gross total resection (GTR) group as reference. To determine an EOR threshold, we created a series of Kaplan-Meier curves at 2% resection intervals, and the first point at which two survival curves intersected was selected as the point of an EOR threshold. Two approaches were used to determine the prognostic value of the variables considered. Firstly, the Cox proportional hazards model (CPHM) was used to determine if the following factors had a statistically significant relationship with survival: age, gender, preoperative tumor volume, postoperative tumor volume, evidence of metastasis, chemotherapy, preoperative Karnofsky Performance Status (KPS), postoperative KPS, epidermal growth factor receptor (EGFR) (+/-), p53 (+/wt), glial fibrillary acidic protein (GFAP) (+/-), isocitrate dehydrogenase (IDH1) (+/-), and Ki-67 index. A forward stepwise selection technique was used to analyze any characteristics associated with survival (p=.05 threshold) in univariate CPHM; these variables were then analyzed using multivariate CPHM. Statistical analysis was performed using JMP 13.1 (SAS Institute, Cary, North Carolina, USA) and Prism 7 (Graphpad Software, La Jolla, California, USA).

## Results

**Figure 1:**
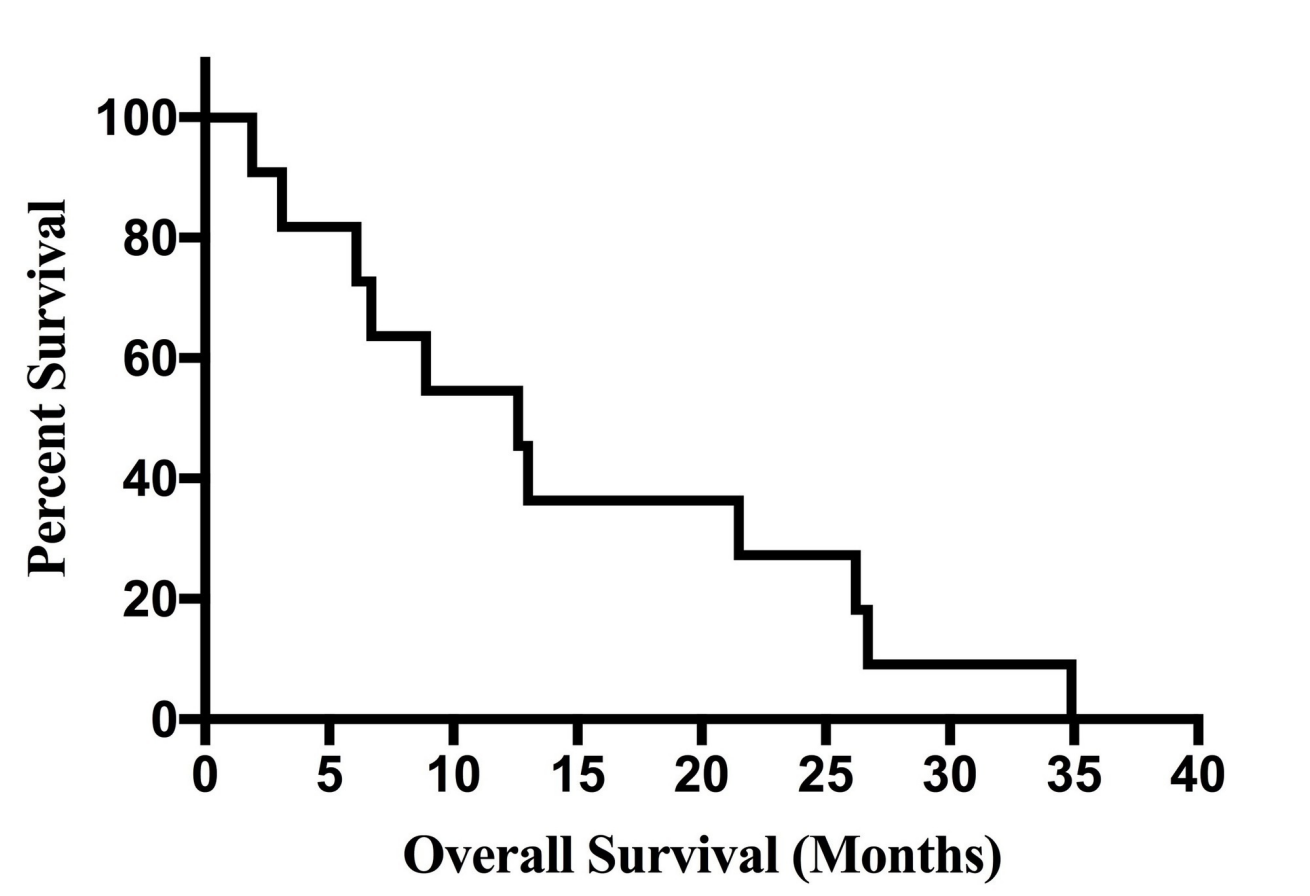
Overall Kaplan-Meier Survival Curve

For the 11 patients diagnosed with GS in this study, overall median survival was 12.6 months (Figure 1). Overall median progression-free survival (PFS) for the group was seven months, but PFS was not found to be a statistically significant predictor of survival. Volumetric analysis showed a median tumor pre-op volume of 28.6 cm^3^, and a median tumor post-op volume of 2.1 cm^3^, both equating to a median extent of resection value of 93% (Table 1). Immunohistochemical data of patient tumors is displayed in Table 2. Patient demographic and clinical details are summarized in Table 3.

**Table 1:** Demographics of Patients in Study Values provided as median (with range) or absolute number (with percentage). Statistical significance at p=.05 level. Karnofsky Performance Status (KPS) scores not available for all patients.

Characteristic		N= 11	P-Value
Age at Diagnosis		63 (54 - 78)	p < .001
Gender	Male	7 (64%)	NS
	Female	4 (36%)	
Preoperative Tumor Size (cm^3^)	1 – 15	1 (9%)	NS
	16 – 30	5 (46%)	
	31 – 45	2 (18%)	
	>45	3 (27%)	
Postoperative Tumor Size (cm^3^)	0 - 2.49	7 (64%)	NS
	2.5 – 5	2 (18%)	
	6 – 10	2 (18%)	
Extent of Resection	> 95%	4 (36%)	REF
	90- 95%	5 (46%)	NS
	< 90 %	2 (18%)	p = .02
Evidence of Metastasis- Intracranial Focality	Unifocal	9 (82%)	p= .03
	Multifocal	2 (18%)	
Evidence of Metastasis- Leptomeningeal Enhancement	Present	2 (18%)	NS
	Absent	9 (82%)	
Evidence of Metastasis- Extracranial Advancement	Present	1 (9 %)	NS
	Absent	10 (91%)	
Chemoradiotherapy	No	1 (9%)	p = .03
	Yes	10 (91 %)	
Preoperative KPS	90	3 (27%)	p =.03
	80	4 (36%)	
	70	2 (18%)	
Postoperative KPS	100	1 (9%)	p = .03
	90	3 (27%)	
	80	3 (27%)	
	70	2 (18%)	

**Table 2:** Immunohistochemical Characteristics Values provided as absolute number with percentage. Immunohistochemical data not available for all patients. EGFR = Epidermal Growth Factor Receptor; P53 = P53 Staining; GFAP = Glial Fibrillary Acidic Protein; IDH1 = Isocitrate Dehydrogenase; KI Index = Antigen Ki-67 Index.

Tumor Characteristic		N=	P-Value
EGFR Staining Intensity	0	2 (18%)	NS
	+1	1 (9%)	
	+2	3 (27%)	
	+3	3 (27 %)	
P53	+	9 (82%)	-
	wt	0 (0%)	
GFAP	+	9 (82%)	NS
	-	1 (9%)	
Mutant IDH1	+	0 (0 %)	-
	-	5 (46%)	
KI Index	< 30 %	5 (46%)	p < .001
	30 – 50 %	2 (18%)	
	≥ 50 %	3 (27%)	

**Table 3:** Patient Characteristics Evidence of metastasis presented as (Focality, Leptomeningeal Enhancement, Extracerebral Spread); 1 = Unifocal, 2 =Multifocal

Patient Number	OS (Months)	Age (Years)	Gender	Pre/Postop Tumor Volume (cm^3^)	EOR	Chemo-RT	Evidence of Metastasis	Pre/Postop KPS
1	8.9	63	M	22.2/.50	.98	+	(1, -, -)	90/90
2	1.9	78	F	28.6/6.1	.79	-	(2, -, -)	70/-
3	13.0	63	M	60.1/2.1	.97	+	(1, -, -)	80/80
4	3.1	78	F	35.5/2.5	.93	+	(1, -, -)	70/70
5	6.1	73	M	82.5/4.7	.94	+	(2, -, -)	-/-
6	21.5	59	M	21.8/1.0	.95	+	(1, +, +)	90/100
7	12.6	65	M	21.1/1.6	.92	+	(1, -, -)	-/80
8	26.2	66	M	26.0/2.1	.92	+	(1, -, -)	80/80
9	34.9	54	M	36.6/0	1.0	+	(1, +, -)	80/90
10	26.7	55	F	65.5/6.6	.90	+	(1, -, -)	90/90
11	6.7	56	F	4.3/1.8	.58	+	(1, -, -)	80/70

**Figure 2:**
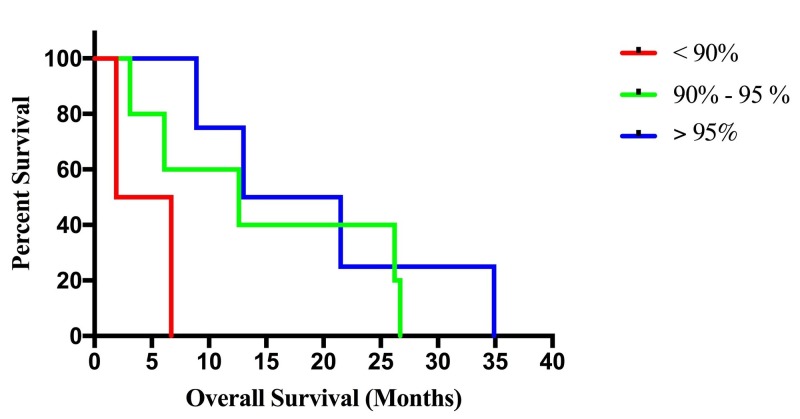
Kaplan-Meier Survival Curves Stratified by Extent of Resection (EOR)

Extent of resection categories were defined as gross total resection (GTR >95%), subtotal resection (STR 90%-95%), and partial resection (PR <90%), and the median overall survival for the groups were as follows: GTR- 17.3 months, STR-12.6 months, PR-4.3 months (Figure 2). There were four patients in the GTR group, five patients in the STR group, and two patients in the PR group. The paired log-rank test confirmed a statistically significant association, at the p=.05 level, between survival and the PR (p=.01) resection group with the GTR group as reference. However, no association was found between survival and the STR group with the GTR group as reference. Univariate CPHM confirmed a statistically significant association between increased survival and age, chemo-radiotherapy, pre-operative KPS, post-operative KPS, focality, and KI Index. Multivariate analysis demonstrated a continued statistical significance for age, preoperative KPS, postoperative KPS, and KI index (Table 4). Serial Kaplan-Meier curves, constructed at 2% EOR intervals, suggest a survival benefit at an EOR threshold of 94%.

**Table 4:** Results of Multivariate Cox Proportional Hazards Model (CPHM) Forward stepwise selected variables from univariate CPHM at the p=.05 level. Multivariate CPHM results displayed, with significance at p=.05 level. Table indicates unit hazard ratios; unit defined as 1 year in age, 10 on Karnofsky Performance Status (KPS) scale, 1% on KI index.

Variable	Unit HR (95% CI)	P-Value
Age	1.21 (.99- 1.58)	p=.05
Tumor Focality	-	NS
Chemoradiotherapy	-	NS
Preoperative KPS	.79 (.48- 1.05)	p = .04
Postoperative KPS	.84 (.59 – 1.08)	p < .01
KI Index	1.02 (.98-1.08)	p < .01

## Discussion

The 2016 updated WHO central nervous system (CNS) guidelines classify tumors by molecular and genetic markers, and group high-grade gliomas by IDH status. GS is classified as an IDH-wildtype variant along with giant cell glioblastoma and epithelioid glioblastoma [3]. Histologically, necrosis and endothelial proliferation must be present for the diagnosis of high-grade glioma, and specifically, for diagnosis of GS, tumors must show both glial and mesenchymal differentiation.

The current standard of care for high-grade gliomas primarily is surgical resection followed by adjuvant chemoradiotherapy with temozolomide. Median survival time is approximately 6-17.5 months across multiple studies (Table 5). Multiple studies have documented a positive association between extent of resection and survival in glioblastoma patients, and one study suggests a threshold of resection for maximum benefit exists at 80% [4]; however, to our knowledge, no study exists that defines an EOR threshold for GS. One study [2] utilizing the Surveillance, Epidemiology, and End Results (SEER) database suggests age, the extent of resection, and use of adjuvant radiotherapy to be associated with increased survival in GS. Determining an appropriate surgical treatment paradigm for GS is difficult given the nature of GS as diffusely infiltrating.

**Table 5:** Case Series Review Examining Studies with Gliosarcoma OS = Overall Survival; GS = Gliosarcoma; TMZ = Temozolomide; RT = Radiotherapy; GSM = Gliosarcoma; GBM = Glioblastoma.

Lead Author	Country	Primary Endpoint	Number of Patients	Overall Survival (Median)	Interventions
Castelli et al. 2016 [1]	France	To identify prognostic or therapeutic variables impacting on OS in GS patients	75	13 Months	Chemoradiotherapy
Adeberg et al. 2016 [5]	Germany	To analyze effect of additional TMZ therapy on GS patients	37	13.4 Months	Chemoradiotherapy
Rath et al. 2015 [6]	India	To analyze the outcome of treating gliosarcoma with RT and TMZ by measuring OS	27	16.7 Months	Chemoradiotherapy
Singh et al. 2015 [7]	India	To analyze the clinical, radiological, histopathological, features of GS treated	16	6 Months	Chemoradiotherapy
Cachia et al. 2015 [8]	United States	To compare the overall survival and pathological features of primary and secondary gliosarcoma	34	17.5 Months	Chemoradiotherapy
Kumar et al. 2015 [9]	India	To evaluate the clinicopathological variables and treatment outcomes in patients of primary GS	27	9 Months	Chemoradiotherapy
Damodaren et al. 2014 [10]	Australia	To review care for GSM patients and compare survival with that of GBM patients	19	9.7 Months	Chemoradiotherapy

Outcomes following GS resection have not been studied as extensively as those in glioblastoma patients due to GS’s lower incidence. Kozak et al.’s [2] SEER study identified 353 GS patients over the age of 20 years in a 17-year period, amounting to an approximate annual incidence of one new case per 10,000,000 people over the age of 20 within the United States. In our study, GS accounted for approximately 2% of all high-grade gliomas treated at the Hospital of the University of Pennsylvania from January 2005 through January 2015. In accordance with previous studies, GS predominantly affected males in our study with a M/F ratio of 1.75:1. The median age of presentation was 63 years old, also similar to previous patient series. Studies have suggested there are statistically significant similar biological features of glioblastoma and GS [11], and our institution has treated both types of patients similarly in the above time period with surgical intervention being the primary step.

Overall, our study showed an increase in survival associated with the gross total resection group in comparison to the PR group. EOR was limited for the two patients in the PR group due to tumor location as one case involved an eloquent region and the other infiltrated across the corpus callosum. No significant difference was found between the STR and GTR group, suggesting no stepwise improvement in survival above a threshold level, which in our study was found to be an EOR of 94%. This may in part be due to the more malignant nature of GS requiring a higher threshold for resection, although this study was not designed to support this theory.

Similar to other findings, our study found adjuvant chemoradiotherapy to be statistically associated with increased survival in univariate analysis. However, in our study, chemoradiotherapy was associated with increased survival during univariate analysis, but when adjusting for multivariate confounders did not maintain significance. This may also be due to the fact that the aggressiveness of GS in relation to glioblastoma makes it more resistant to adjunct treatment following surgical resection.

Studies have reported GS as having a greater propensity to metastasis [12-13] outside of the brain and this is believed to be due to the sarcomatous character of the tumor to spread hematogenously. Our series contained two (18%) patients with multifocal lesions, and while multifocal lesions were statistically associated with differing survival in our study at the univariate level, it did not maintain significance in the multivariable analysis. It is plausible that the multifocal lesions are demonstrative of concomitant gliomas, which has been studied in one glioblastoma study [14], but to our knowledge, no such study has been completed for GS. However, given the aggressive nature of GS and its sarcomatous component, it is likely that these multifocal enhancing portions were part of the same infiltrating neoplasm since immunohistochemistry staining suggested a similar origin. In addition, our study included two (18%) patients with leptomeningeal enhancement and one (9%) patient with extracerebral metastasis.

As in other studies, both preoperative and postoperative KPS scores were associated with increased survival. In our study, two patients had improved performance postoperatively, while five patients remained at the same functional level postoperatively. However, there was no significant improvement in KPS scores recorded in association with the gross total resection category with this analysis, limited by the overall small sample size.

A lack of EGFR amplification in GS is a molecular marker that makes it distinct from glioblastoma. The frequency of EGFR amplification is known to be low in GS and is reported to be present in approximately 8% of cases [15-16]. Our study used immunohistochemistry staining to measure EGFR expression (scale 0-3), and three samples demonstrated strong (+3) immunohistochemical expression; however, genetic profiling would be required to determine amplification status. In our study, no association was found between the results of EGFR staining and survival. Additionally, the Ki-67 index, a molecular marker for cell proliferation, demonstrated an association with decreased survival in this study.

The authors certainly note the limitations of this study as a retrospective cohort study of a specific population subset at one academic institution. It is important to note that the patients in the study differed in multiple clinical factors; however, our study agrees with overwhelming evidence in the study of high-grade gliomas that extent of resection is associated with increased survival. We also note our limited sample size and the biases from differences in patient characteristics that can influence results. Despite these limitations, our patient profiles were well indexed and thus we were able to analyze many clinical and immunohistochemistry factors associated with the tumor’s outcomes. Additional studies will be necessary to determine the optimal balance between surgical and post-operative treatment for maximal survival in GS patients.

## Conclusions

This study supports preexisting evidence from glioblastoma that there is an association between extent of resection and survival, and it suggests that this association is valid in GS patients. Our findings demonstrate that an EOR >94% resection is associated with increased survival. Therefore, achieving an extent of resection above this threshold should be a priority for neurosurgeons treating patients with GS. In addition to maximum resection of the enhancing portion of the lesion, age (inversely), preoperative KPS, and postoperative KPS index are associated with lengthened survival and should be considered in determining a treatment plan for GS patients when possible.
